# Humoral and cellular responses to a non-adjuvanted monovalent H1N1 pandemic influenza vaccine in hospital employees

**DOI:** 10.1186/1471-2334-13-544

**Published:** 2013-11-15

**Authors:** Ma Teresa Herrera, Yolanda Gonzalez, Esmeralda Juárez, Fernando Hernández-Sánchez, Claudia Carranza, Carmen Sarabia, Silvia Guzman-Beltran, Ma Eugenia Manjarrez, Marcela Muñoz-Torrico, Lourdes Garcia-Garcia, Eduardo Sada, Martha Torres

**Affiliations:** 1 Department of Microbiology, Instituto Nacional de Enfermedades Respiratorias Ismael Cosío Villegas, Tlalpan 4502, Tlalpan, Mexico City 14080, Mexico; 2 Department of Virology, Instituto Nacional de Enfermedades Respiratorias Ismael Cosío Villegas, Tlalpan 4502, Tlalpan, Mexico City 14080, Mexico; 3 Centro de Investigacion sobre Enfermedades Infecciosas, Instituto Nacional de Salud Publica, Cuernavaca, Mexico

**Keywords:** Pandemic influenza, H1N1, Vaccine, Cellular response, Proliferation, Humoral response

## Abstract

**Background:**

The efficacy of the H1N1 influenza vaccine relies on the induction of both humoral and cellular responses. This study evaluated the humoral and cellular responses to a monovalent non-adjuvanted pandemic influenza A/H1N1 vaccine in occupationally exposed subjects who were previously vaccinated with a seasonal vaccine.

**Methods:**

Sixty healthy workers from a respiratory disease hospital were recruited. Sera and peripheral blood mononuclear cells (PBMCs) were obtained prior to and 1 month after vaccination with a non-adjuvanted monovalent 2009 H1N1 vaccine (Influenza A (H1N1) 2009 Monovalent Vaccine Panenza, Sanofi Pasteur). Antibody titers against the pandemic A/H1N1 influenza virus were measured via hemagglutination inhibition (HI) and microneutralization assays. Antibodies against the seasonal HA1 were assessed by ELISA. The frequency of IFN-γ-producing cells as well as CD4^+^ and CD8^+^ T cell proliferation specific to the pandemic virus A/H1N peptides, seasonal H1N1 peptides and seasonal H3N2 peptides were assessed using ELISPOT and flow cytometry.

**Results:**

At baseline, 6.7% of the subjects had seroprotective antibody titers. The seroconversion rate was 48.3%, and the seroprotection rate was 66.7%. The geometric mean titers (GMTs) were significantly increased (from 6.8 to 64.9, p < 0.05). Forty-nine percent of the subjects had basal levels of specific IFN-γ-producing T cells to the pandemic A/H1N1 peptides that were unchanged post-vaccination. CD4^+^ T cell proliferation in response to specific pandemic A/H1N1 virus peptides was also unchanged; in contrast, the antigen-specific proliferation of CD8^+^ T cells significantly increased post-vaccination.

**Conclusion:**

Our results indicate that a cellular immune response that is cross-reactive to pandemic influenza antigens may be present in populations exposed to the circulating seasonal influenza virus prior to pandemic or seasonal vaccination. Additionally, we found that the pandemic vaccine induced a significant increase in CD8^+^ T cell proliferation.

## Background

In the spring of 2009, the most recent pandemic of the A/H1N1 influenza virus originated in Mexico, eventually becoming a major worldwide public health threat [1]. With the goal of protecting high-risk populations, different vaccine formulations were produced that yielded high seroprotection and seroconversion rates in healthy subjects; high antibody titers were observed beginning on day 10 after vaccination and were sustained up to day 45 [2-4]. Mexican health authorities began by administering the vaccine to high-risk groups including hospital employees who had previously received the seasonal influenza vaccine. The employees of the National Institute of Respiratory Diseases in Mexico City were vaccinated with the non-adjuvanted monovalent pandemic influenza A/H1N1 vaccine manufactured by Sanofi Pasteur, a vaccine that was based on the virus strain recommended by the World Health Organization for the development of the influenza A (H1N1) vaccine [5]. The antibody response to the Sanofi Pasteur vaccine has been reported to range from good to moderate in different high-risk populations [6-9]. Nevertheless, our knowledge regarding the induction of cellular responses to this vaccine remains limited.

In addition to a humoral response, this vaccine is expected to promote the development of cell-mediated immune responses [10]. Cellular immune responses, mediated by CD4^+^ and CD8^+^ T cells, contribute to essential surveillance against the influenza virus via cytokine modulation and the cross-reactivity that exists among virus-specific cytotoxic T cells [11]. Moreover, the use of the IFN-γ ELISPOT assay has successfully demonstrated the existence of circulating memory CD4^+^ and CD8^+^ T cells that are cross-reactive with the pandemic virus in healthy donors irrespective of any prior seasonal vaccination or exposure to this novel virus [11-14].

The present study was initiated after the first pandemic wave in Mexico occurred and was conducted as a longitudinal study in employees of the National Institute of Respiratory Diseases in Mexico City. This study was conducted to evaluate the immunogenicity of a single-dose administration of a monovalent non-adjuvanted pandemic influenza A/H1N1 vaccine in individuals who had previously received the trivalent 2008 or 2009 seasonal influenza vaccine. We evaluated the level of protection of the pandemic A/H1N1 vaccine according to specific antibody titers as well as human influenza-specific T cell responses.

## Methods

### Study group

We recruited 60 healthy subjects at random who intended to receive the pandemic influenza vaccine through a public institutional vaccine program for employees of the National Institute of Respiratory Diseases in Mexico City; of these subjects, 45 (75%) had received the 2009 seasonal vaccine (Fluzone 2009–2010 trivalent A/Brisbane/59/2007 (H1N1)-like, A Brisbane/10/2007 (H3N2)-like and B/Brisbane/60/2008-like strains) in October 2009, and 5 subjects (8.3%) had received the 2008 trivalent seasonal vaccine (Fluzone 2008–2009 A/Solomon Islands/3/2006 (H1N1), A Wisconsin /67/2005 (H3N2) B Malaysia/2506/2004). The authors did not vaccinate the cohort, and this was not a trial study. The subjects were recruited from December 2009 to January 2010. All volunteers received a single dose of the non-adjuvanted monovalent 2009 H1N1 vaccine (Influenza A (H1N1) 2009 Monovalent Vaccine Panenza, Sanofi Pasteur). The volunteers consented to undergo venipuncture prior to and 1month after pandemic influenza A/H1N1 vaccination. We obtained paired samples for the isolation of serum and peripheral blood mononuclear cells (PBMCs). All participants signed an informed consent form, and the local Institutional Review Boards approved this protocol.

### Peptides

We used custom-designed peptides that were synthesized by Enzo Life Sciences (Ann Arbor, MI, U.S.A.) and designed on the basis of their conservation level for viral strain discrimination. The regions of the hemagglutinin (HA) sequences of the 2009 pandemic H1N1 and seasonal H1N1 and H3N2 viruses that were unique to these viruses were selected and analyzed. All HA sequences from the National Center for Biotechnology Information (NCBI) Influenza Virus Sequence Database available in 2009 were downloaded and aligned using the ClustalW (FASTA) tool, which is free and available on the NCBI website [15]. Protein consensus sequences were generated and independently analyzed for each virus subtype; the scores for conservation or synonymous substitutions were identified for each position. The selected sequences contained putative MHC class I and class II epitopes with low and high affinity for HLA alleles that are frequently found in the Mexican population [16]. These sequences were validated with the SYFPEITHI epitope prediction tool and the ProPred MHC class II binding prediction server [17] (see Additional file 1: Table S1).

Lyophilized peptides were dissolved in RPMI 1640 medium (Lonza, Walkersville, MD) containing 20% dimethyl sulfoxide (DMSO, Sigma-Aldrich, St Louis, MO), frozen at -70°C and diluted at a ratio of 1:1,000 with RPMI 1640 medium containing 2 mM L-glutamine (Sigma-Aldrich) and 50 μg/ml gentamicin sulfate (Lonza), herein termed “supplemented medium”.

### Sera and cells

After blood samples were collected from the volunteers, serum was obtained via centrifugation and stored at -70°C until use. PBMCs were isolated from heparinized blood diluted at a 1:2 ratio with supplemented medium and separated via centrifugation using a lymphocyte separation solution (Lonza). The PBMCs were washed, counted and cryopreserved in fetal bovine serum (FBS, Hyclone, Logan, UT) containing 10% DMSO at -70°C until use. For the cellular response analysis, cryopreserved PBMCs were thawed and re-suspended in supplemented medium (viability ≥ 90%).

### Antibody detection

Antibodies specific to the influenza pandemic virus were identified using hemagglutination inhibition and microneutralization assays. Briefly, serial 2-fold dilutions of the previously inactivated serum were assayed for their ability to inhibit the agglutination of chicken red blood cells in the presence of the A/H1N1/California/07/2009 influenza virus. Serum dilutions (starting at 1:5) were tested in duplicate, and titers are expressed as the reciprocal of the highest dilution that produced complete hemagglutination or 50% neutralization, respectively [18]. The antibody response was assessed using the criteria of the European Agency for the Evaluation of Medicinal Products (EMA) for 18- to 60-year-old subjects and was based on the geometric mean titers (GMTs), seroprotection rate (percentage of subjects with HI antibody titers ≥ 1:40) and seroconversion rate (percentages of subjects with either pre-vaccination HI antibody titers ≤ 1:10 and post-vaccination titers ≥ 1:40 or pre-vaccination HI antibody titers ≥ 10 and at least a 4-fold increase in post-vaccination HI antibody titers). In accordance with the EMA, these criteria include a seroprotection rate ≥ 70%, a seroconversion rate ≥ 40% and a GMT fold increase of 2.5; these 3 parameters must be fulfilled for acceptable pandemic influenza vaccines [19]. We also determined the levels of anti-H1 hemagglutinin (HA1) IgG antibodies in the serum using a commercial ELISA kit (Cusabio, Biotech Co., Newark, DE) according to the manufacturer’s instructions. The results are expressed as the OD at 450 nm. A sample was considered seropositive when its OD was ≥ 0.4.

### Human IFN-γ ELISPOT assay

The frequency of antigen-specific IFN-**γ**-producing T cells upon stimulation with HA peptides was assessed using ELISPOT, as previously described [20,21]. In brief, multiscreen 96-well filter plates (Millipore Corporation, Billerica, MA) were incubated overnight at 4°C with anti-human IFN-**γ** antibody (Endogen, Woburn, MA). The plates were blocked with 1% bovine serum albumin (BSA, Calbiochem, La Jolla, CA), and 2.5 × 10^5^ PBMCs/well were seeded and incubated for 48 h with individual peptides (10 μg/ml), a mix of peptides (2 μg/ml each) or the recombinant HA A/H1N1/California/06/2009 (Immune Tech., Foster City, CA). Phytohemagglutinin (10 μg/ml) (PHA, Sigma-Aldrich) was included as the positive control, and the medium alone culture served as the negative control (background). The cells were removed, and biotinylated-anti-human IFN-**γ** antibody (Endogen) was added and allowed to incubate for 2 h. Peroxidase-streptavidin (Dako, Glostrup, Denmark) was added, and the spots were visualized after the addition of 1% 3-amino-9-ethylcarbazole (AEC, Pierce, Rockford, IL). Spot-forming cells (SFCs) were counted using an ImmunoSpot reader (Cellular Technology Ltd, Cleveland, OH). The frequency of IFN-γ-producing cells was calculated by averaging the number of spots in duplicate wells after background subtraction. The results are expressed as SFCs/10^6^ PBMCs. Subjects whose IFN-γ response exceeded the background response by at least 6 spots were considered specific responders.

### T cell proliferation assay

To evaluate the proliferative response after pandemic A/H1N1 vaccination, we examined the percentage of proliferating peripheral CD4^+^ and CD8^+^ T cells following stimulation with H1N1 peptides. This analysis utilized the carboxyfluorescein diacetate succinimidyl ester (CFSE) dilution model, in which the CFSE signal is divided between daughter cells and shows peaks with diminishing fluorescence at an approximate ratio of 0.5 per cell division. We analyzed each round of cell division of CD8^+^ and CD4^+^ T cells in response to the H1N1 peptides. Briefly, the PBMCs were labeled with CFSE (Sigma-Aldrich), placed in a PBS/2% BSA solution for 15 min at 37°C and washed twice with PBS. The cells were then seeded in ultralow attachment 24-well plates (Costar-Corning Incorporated, Corning, NY) at 1 × 10^6^ PBMCs/well and stimulated with 10 μg/ml of the individual influenza peptides. PHA (10 μg/ml) was included as a positive control, and medium alone served as the negative control. The plates were incubated at 37°C with 5% CO_2_ for 7 days. The cells were subsequently stained with monoclonal anti-human antibodies including CD3-PE-Texas Red (Invitrogen Gibco, Grand Island, NY), CD8-PE-Cy7 and CD4-APC-Cy7 (BioLegend, San Diego, CA). Then, 1 × 10^4^ events were recorded for each condition using a FACSCanto II flow cytometer (Becton Dickinson), and the data were analyzed with the FlowJo proliferation platform (FlowJo 9.4.10, Tree Star, Inc., Ashland, OR). The peptide-specific proliferation of CD3^+^/CD8^+^/CFSE^+^ or CD3^+^/CD4^+^/CFSE^+^ cells was calculated by subtracting the number of proliferating cells in the negative control. Individuals with a positive proliferative response were defined as subjects with ≥ 2% proliferating cells (based on the summed responses to peptides 1 and 2 and the total proliferation).

### Statistical methods

Differences in antibody production and IFN-γ responses elicited by the specific peptides and the proliferation of CD8^+^ and CD4^+^ T cells pre- and post-vaccination were analyzed using the Wilcoxon signed rank test. Differences in proliferation between the influenza serotypes were analyzed using the Kruskal-Wallis test, followed by Dunn's multiple comparison test. Associations of age, gender and area of work at the hospital with pre- and post-vaccination antibody titers were assessed using the chi-square test. We used the Spearman rank correlation test to assess whether age, gender or work activity at the hospital was associated with neutralizing antibody titers or T cell responses. To our knowledge there are no published data describing the mean difference in CD8^+^ T cells between pre- and post-H1N1 pandemic influenza vaccination. We therefore estimated our sample size based on an analysis of the first 10 pairs of subjects among whom we observed that the difference in response was not normally distributed (median 3.8, IQR (1.44-7.09). We calculated the sample size needed for a two-sample t-test (alpha = 0.05 and beta = 0.8) using a mean of 3.8 and standard deviation of 4, arriving at 33 pairs, and then adjusted the sample size based on the asymptotic relative efficiency of the Mann–Whitney U relative to the t-test (worst case scenario, dividing 33 by 0.864), arriving at 38 pairs [22].

The statistical analyses were performed using SPSS 15.0 for Windows (SPSS, Chicago, IL) and Prism 5.0 (GraphPad, La Jolla, CA). Two-tailed p-values < 0.05 were considered statistically significant.

## Results and discussion

### Description of the cohort

Sixty subjects were enrolled in the present study; all of the subjects were employees of the National Institute of Respiratory Diseases in Mexico City, which is a reference center for pandemic influenza patients. All subjects were actively working during the pandemic wave of the influenza A/H1N1 virus (2009), which originated in Mexico. Forty-five (75%) of the subjects were previously vaccinated with the trivalent influenza seasonal vaccine in October 2009, and 5 (8.3%) of the subjects were vaccinated with the 2008 trivalent influenza seasonal vaccine before October 2009. The occupations of the participants included research, clinical practice and administrative functions. None of the subjects had been diagnosed as having influenza prior to or after A/H1N1 vaccination. Table 1 summarizes the characteristics of the study group. The variables of age, gender, prior seasonal vaccination status and area of work were not associated or correlated with the humoral or cellular responses described below.

**Table 1:** Demographics of the study group

	**Value**
N	60
Female	58%
Male	42%
Age	37.1 ± 8.8
Immunization with 2008–2009 seasonal vaccine	8.3%
Immunization with 2009–2010 seasonal vaccine	75.0%
No prior seasonal vaccination	16.7%
Work-related direct contact with patients	21.1%

### Antibody responses to the pandemic A/H1N1 vaccine

**Figure 1:**
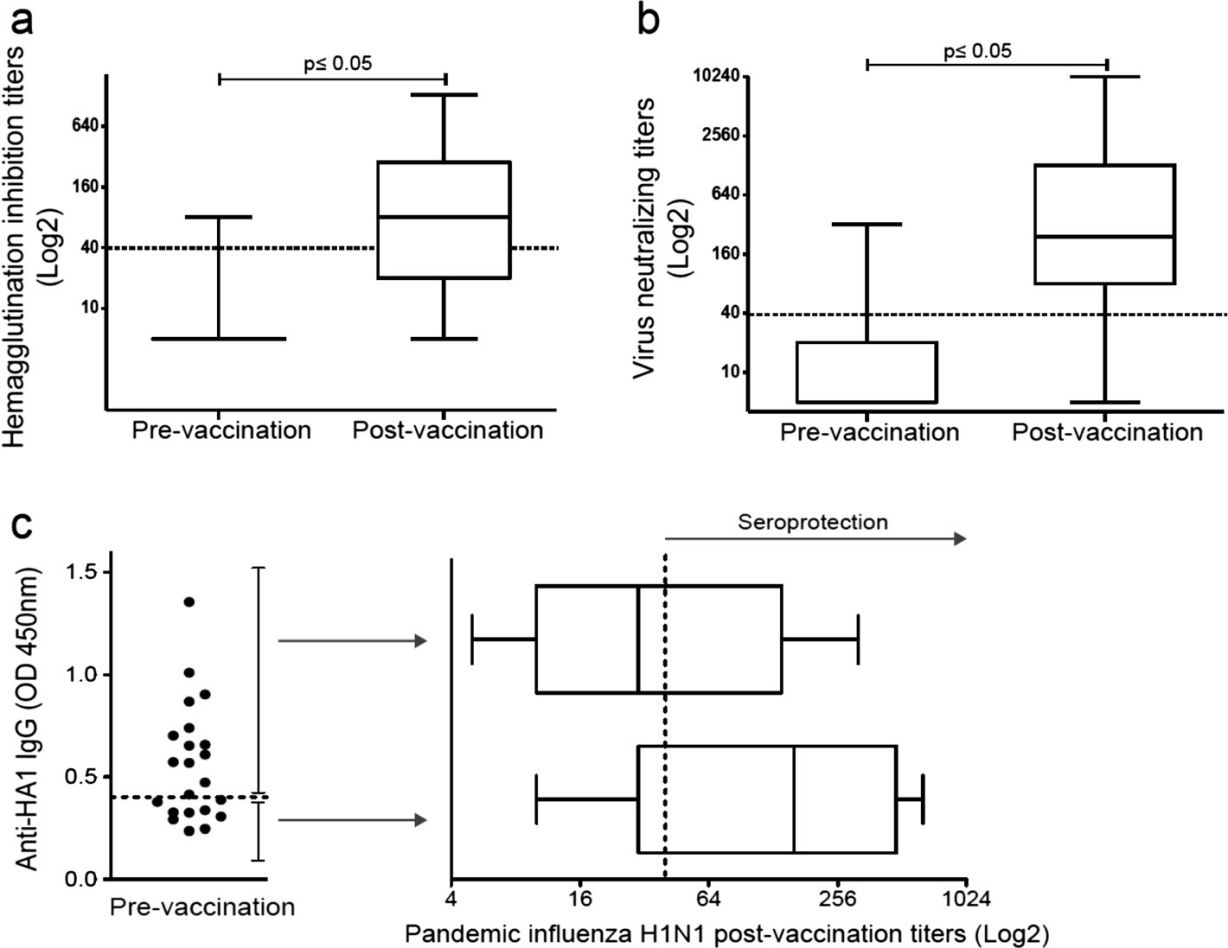
**Increased humoral responses after pandemic influenza A/H1N1 vaccination.** Specific antibodies against the pandemic influenza virus A/H1N1 were measured via **(a)** hemagglutination inhibition and **(b)** microneutralization. The box plots show quartiles and medians. The dotted lines indicate a cut-off value of 1:40, with p ≤ 0.05 (pre-vaccination vs. post-vaccination), as determined using the Wilcoxon singed rank test (n = 60). **(c)** The presence of IgG antibodies against hemagglutinin A/H1N1 (HA1) was assessed in sera obtained prior to H1N1 vaccination using ELISA, with the dotted line indicating the cut-off value (n = 22). The antibody titers against the pandemic influenza virus A/H1N1 were assessed post-vaccination in sera from individuals with (n = 13) or without (n = 9) anti-HA1 antibodies prior to vaccination using the hemagglutination inhibition assay. The depicted box plots show the quartiles and medians. The dotted line indicates the protective cut-off value.

The immunogenicity of the vaccine against influenza A/H1N1 was assessed according to the antibody titer after vaccination. Here, we determined the serum levels of antibodies against the pandemic virus using a hemagglutination inhibition assay, which is a standard assay used in the process of licensing influenza vaccines. Prior to 2009 A/H1N1 virus vaccination, the baseline seroprotective rate of the subjects was 6.7%, and the GMT was 6.8 (Figure 1a and Table 2). The proportion of subjects with titers ≥ 1:40 in the hemagglutination inhibition assay at baseline was lower than expected for such an exposed population considering the previous vaccination with the 2008 and 2009 seasonal influenza vaccines in 83% of the subjects; these titers were also lower than those previously reported for health care workers [6]. Antibodies are expected to be present in healthy subjects prior to vaccination with the pandemic A/H1N1 vaccine [23,24] and are likely elicited by natural exposure to cross-reactive influenza strains [25].

**Table 2:** Humoral response after pandemic H1N1 vaccination

	**Hemagglutination inhibition**		**Microneutralization assays**	
	***Pre-vaccination***	***Post-vaccination***	***Pre-vaccination***	***Post-vaccination***
N	60	60	60	60
Seroprotection rate%^a^	6.7	66.7^d^	NA	NA
Seroconversion rate%^b^	NA	48.3	NA	NA
GMT^c^	6.8	64.9^d^	10.7	288.4^d^
GMT Fold-change	NA	9.5	NA	26.9

^a^Seroprotection rate = percentage of subjects with hemagglutinin inhibition titers ≥ 1:40.

^b^Seroconversion rate = percentage of subjects with pre-vaccination hemagglutinin inhibition titers ≤ 1:10 and post-vaccination titers ≥ 1:40.

^c^GMT = geometric mean titer.

^d^p ≤ 0.05 vs pre-vaccination.

After vaccination, the seroconversion (48.3%) and seroprotection (66.7%) rates increased, as did the antibody GMTs (p < 0.0001). Although pandemic influenza vaccination induced a humoral response with a high fold increase in the GMT (Table 2), our study most likely lacked sufficient power to detect the seroprotection rate required for vaccination success according to the international criteria [19].

Furthermore, we performed a virus microneutralization assay and observed a significant increase in the virus neutralizing antibody titers after vaccination (p ≤ 0.0001, Figure 1b). Although there are no standardized criteria to apply for this method, our results showed that pandemic influenza vaccination induced a limited humoral response in this study group.

It has been reported that recent seasonal vaccination can decrease antibody responses to the pandemic A/H1N1 vaccine [6,26,27], but the mechanism of such a phenomenon has not been explored. We investigated whether the pre-existence of anti-HA1 antibodies played a role in the decrease of the pandemic A/H1N1 vaccine antibody responses in a subgroup of our study cohort that included subjects who were seronegative for the specific pandemic antibodies and were vaccinated with the seasonal 2009 vaccine. We divided this group into individuals who presented anti-HA1 antibodies prior to pandemic A/H1N1 vaccination and individuals who did not. Our results showed that after pandemic A/H1N1 vaccination, the subjects without anti-HA1 antibodies prior to vaccination (n = 9) showed higher hemagglutination inhibition antibody titers compared with subjects with anti-HA1 antibodies prior to vaccination (n = 13) (GMT 108.9 vs. GMT 37.75, respectively, p = 0.14). However, this difference was not statistically significant. Thus, we cannot conclude that pre-existing antibodies to the seasonal vaccine played a role in the induction of antibodies against the pandemic A/H1N1 vaccine (Figure 1c).

### T cell responses to pandemic influenza peptides

#### IFN-γ production

**Figure 2:**
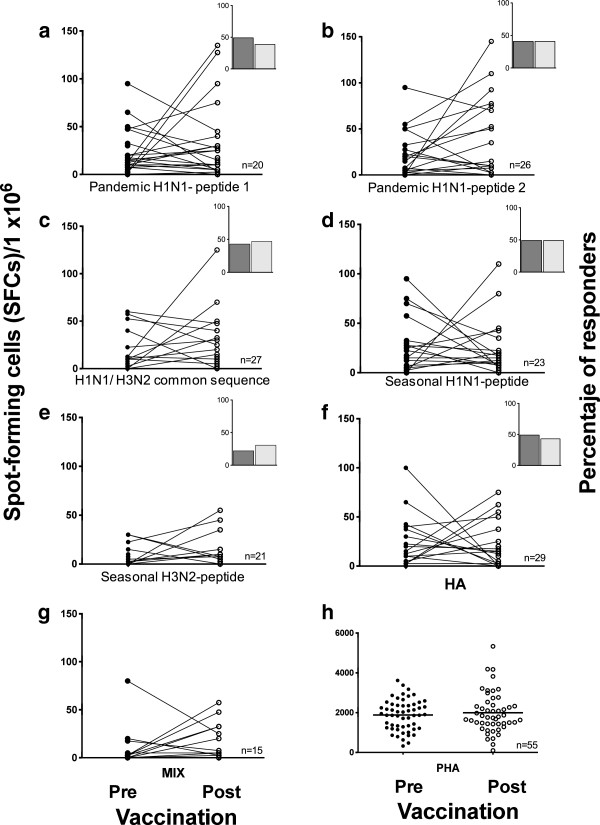
**Frequency of IFNγ-producing cells in response to pandemic influenza peptides.** Pre- and post-vaccination PBMCs were stimulated with 10 μg/ml of pandemic H1N1 peptides 1 and 2, a common H1N1/H3N2 sequence, the seasonal H1N1 and H3N2 peptides **(a-e)**, the whole HA protein **(f)**, a mixture of all the peptides **(g)** or PHA **(h)** for 48 h. Cells producing IFN-γ were enumerated using an ex-vivo ELISPOT assay. The results are expressed as the number of SFCs per 10^6^ PBMCs. Insets depict the percentage of subjects with a positive IFN-**γ** response pre- (dark) and post-(light) vaccination.

To evaluate vaccine-induced T cell reactivity, we assessed IFN-γ production using PBMCs stimulated *in vitro* with specific peptides from pandemic A/H1N1 and the seasonal virus using ELISPOT. The baseline frequency of the IFN-γ-producing cells was moderate and comparable to that reported by others [28,29]. Prior to vaccination, a specific response to viral serotype peptides was observed in a number of subjects and ranged from 22% for the seasonal H3N2 peptide to 49% for the pandemic A/H1N1 peptide 1 (Figure 2). Unexpectedly, after vaccination, IFN-γ production did not increase in response to the specific pandemic A/H1N1 peptides or to peptides from the seasonal strains.

There is controversy in the published data regarding the specifics of IFN-γ production. For example, it has been reported that only CD4^+^ and not CD8^+^ T cells produce IFN-γ in response to specific peptides after vaccination and that this cellular response is low but steady [30]. In contrast, another study reported that subjects without confirmed exposure to the pandemic A/H1N1 virus could generate higher numbers of IFN-γ spots from both CD4^+^ and CD8^+^ T cells to either pandemic A/H1N1 infection with the whole vaccine virus or toward a peptide pool [31,32]. In any case, no correlation was observed regarding antibody production in these studies; we also failed to detect such a correlation in our study. In light of these findings, our observation of the absence of a post-vaccination response was unexpected and may be due to the selectivity of the immune response of antigen-specific cells that can produce cell mediators, such as cytotoxic effectors, in addition to IFN-γ [11].

#### CD4^+^ and CD8^+^ T cell proliferative responses

**Figure 3:**
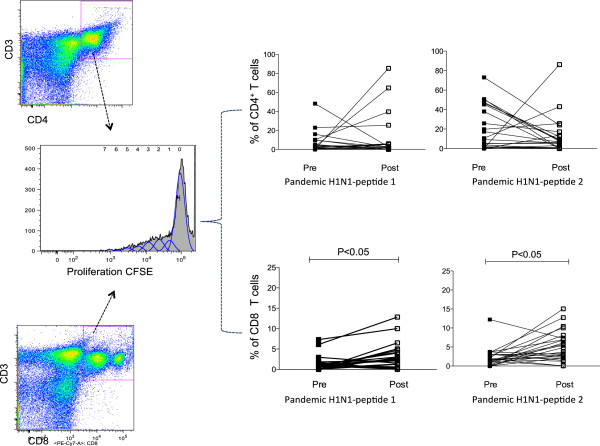
**Proliferative T cell responses to pandemic H1N1 and seasonal peptides.** CFSE-labeled PBMCs (1 × 10^6^) were stimulated with 10 μg/ml of pandemic A/H1N1 peptides 1 and 2 for 7 days. The cells were stained with anti-human CD3PE-Texas Red, CD8PE-Cy7 and CD4APC-Cy7 and then analyzed using flow cytometry. The plots show the percentages of proliferating CD4^+^ or CD8^+^ T cells prior to and after A/H1N1 vaccination (n = 32).

Prior to H1N1 vaccination, we observed proliferative responses in peripheral CD4^+^ and CD8^+^ T cells to pandemic A/H1N1 peptides 1 and 2. Following A/H1N1 vaccination, the proportion of proliferative CD8^+^ T cells significantly increased (p < 0.05); however, the proliferation of CD4^+^ T cells did not significantly increase (p = 0.149) (Figure 3). We do not believe that the lack of CD4^+^ T cell proliferation was associated with limited class II peptide recognition by these cells because the selected A/H1N1 peptides (1 and 2) are predicted to have a high affinity for class I and class II recognition in the Mexican population (Additional file 1: Table S1). In the case of proliferative responses to H1N1 peptide 2, a decreasing trend was observed in CD4^+^ T cell proliferation, although this was not significant (p = 0.45) (Figure 3).

It is possible that the lack of a significant increase in CD4^+^ T cell proliferation after H1N1 vaccination may be due to a higher frequency of CD4^+^ T cells compared with CD8^+^ T cells prior to vaccination, which in turn could be induced by seasonal vaccination and/or natural exposure to the influenza virus. It is also possible that the absence of an adjuvant in the vaccine affected the proliferation of CD4^+^ T cells. Similar to our results, a previous report demonstrated that a non-adjuvanted H5N1 vaccine did not induce a robust expansion of CD4^+^ T cells and failed to increase specific antibody titers [33]. Therefore, our results indicate that either the A/H1N1 vaccine had low CD4^+^ T cell immunogenicity or that the A/H1N1 vaccine did not induce CD4^+^ T cell proliferation in individuals who had previously received the seasonal vaccine. In contrast to the CD4^+^ T cell responses, our results indicated that the A/H1N1 vaccine induced significant CD8^+^ T cell proliferation in individuals who had previously received the seasonal vaccine. Additionally, we did not find a correlation between CD4^+^ or CD8^+^ T cell proliferation and the antibody production induced by vaccination.

In summary, our results indicate that pandemic vaccination induces CD8^+^ T cell clonal expansion, as demonstrated by the increase in the proliferative response to A/H1N1 peptides. This result is consistent with previous reports demonstrating that CD8^+^ T cells proliferate after influenza vaccination [28,31]. We hypothesize that the extent of CD8^+^ T cell clonal expansion that we observed following H1N1 pandemic vaccination may be linked to the cytolytic effector functions of cells rather than the generation of antigen-specific long-lived plasma cells and the direct effector functions mediated by IFN-γ [34].

**Figure 4:**
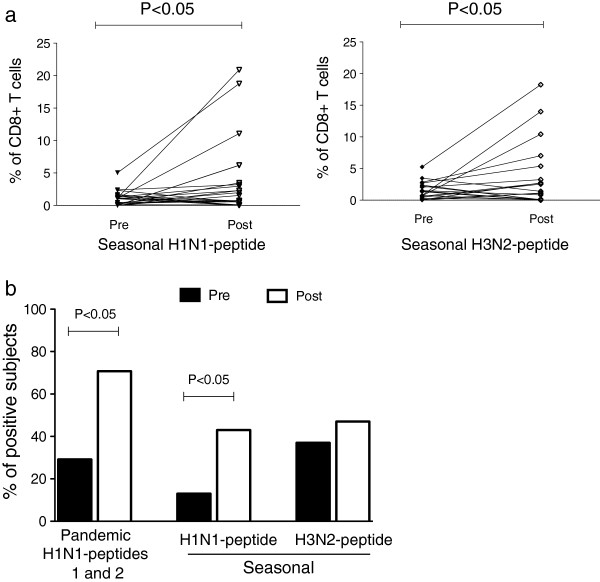
**Pandemic H1N1 vaccination induced CD8**^**+ **^**T cell proliferation to pandemic H1N1, seasonal H1N1 and H3N2 peptides.** CFSE-labeled PBMCs (1 × 10^6^) were stimulated with 10 μg/ml of the pandemic H1N1 and seasonal H1N1 and H3N2 peptides for 7 days. The cells were harvested and stained with anti-human CD3PE-Texas Red and CD8PE-Cy7 and analyzed using flow cytometry. **(a)** Percentages of the CD8^+^ T cell proliferation pre-vaccination and post-vaccination in response to seasonal H1N1 and H3N2 peptides. The means and standard errors are shown, with p ≤ 0.05 pre-vaccination vs. post-vaccination, according to the Wilcoxon matched-pairs signed rank test. **(b)** Percentages of positive subjects responding to the pandemic H1N1 peptides 1 and 2 (p < 0.05) and the seasonal influenza A H1N1 (p < 0.05) and H3N2 serotypes post-vaccination (p > 0.05).

Furthermore, to analyze the cross-reactivity of seasonal serotypes, we evaluated the percentages of CD8^+^ T cells proliferating in response to specific peptides corresponding to seasonal H1N1 and H3N2 serotypes. We found that approximately 5% of the CD8^+^ T cells proliferated in response to pandemic and seasonal peptides prior to vaccination (Figure 4a). This finding may be associated with previous seasonal vaccination because most of the individuals had received a seasonal vaccination approximately 2 months prior to the pandemic vaccination. However, this finding could also be associated with the presence of cross-reactive virus-specific CD8^+^ memory T cells, as reported by others [35]. Our results also showed a significant increase in CD8^+^ T cell proliferation in response to pandemic and seasonal influenza-specific peptides after pandemic A/H1N1 vaccination (p < 0.05) (Figures 3 and 4a). No significant differences were detected in the percentage of proliferating CD8^+^ T cells between the pandemic and seasonal type A viruses. Among individuals with positive proliferative responses, 29%, 13% and 37% had pre-existing CD8^+^ T cells specific to the pandemic A/H1N1, seasonal H1N1 and seasonal H3N2 peptides, respectively. One month after pandemic vaccination, the percentage of positive subjects increased to 70.8% (p < 0.05) for the pandemic A/H1N1 serotype and to 43% (p < 0.05) and 47% (p > 0.05) for the seasonal H1N1 and H3N2 serotypes, respectively (Figure 4b). These data indicate that after pandemic A/H1N1 vaccination, the number of individuals with antigen-specific proliferating CD8^+^ T cells increased; this assessment may serve as a complementary parameter for evaluating the immunological success of influenza vaccines.

## Conclusions

Our results showed that cellular immune responses that are cross-reactive to pandemic influenza antigens may be present in populations exposed to the circulating seasonal influenza virus prior to pandemic or seasonal vaccination. The pandemic vaccine induced a significant increase in CD8^+^ T cell proliferation.

## Abbreviations

HA: Hemagglutinin; PBMCs: Peripheral blood mononuclear cells; ELISPOT: Enzyme-linked-Immuno-Spot; SFC: Spot-forming cell.

## Competing interests

The authors declare that they have no competing interests.

## Authors’ contributions

MTH, FHS and CC performed the experiments, data analysis and results interpretation. MEM and CS performed the data analysis. SGB designed the peptide sequences. MMT performed the clinical analysis. EJ and YG made valuable contributions to the study’s design, analysis and interpretation of the data and the written manuscript. ES and LGG participated in the study design and interpretation of the data. MT participated in the study design and wrote the manuscript. All authors reviewed and approved the final manuscript.

## Authors’ information

Eduardo Sada and Martha Torres, are authors share senior authorship.

## Pre-publication history

The pre-publication history for this paper can be accessed here:

http://www.biomedcentral.com/1471-2334/13/544/prepub

## Supplementary Material

Additional file 1: Table S1 Peptide sequences and predicted recognition by class I and class II HLA alleles frequently found in Mexicans. Click here for file
