# Supplementary material for: Humoral and cellular responses to a non-adjuvanted monovalent H1N1 pandemic influenza vaccine in hospital employees
Source: BMC Infect Dis. 2013 Nov 15;13:544. doi: 10.1186/1471-2334-13-544 (PMC3835617; doi:10.1186/1471-2334-13-544)
Supplement: Additional file 1: Table S1 — Peptide sequences and predicted recognition by class I and class II HLA alleles frequently found in Mexicans. [file 1471-2334-13-544-S1.pptx]

## Slide 1
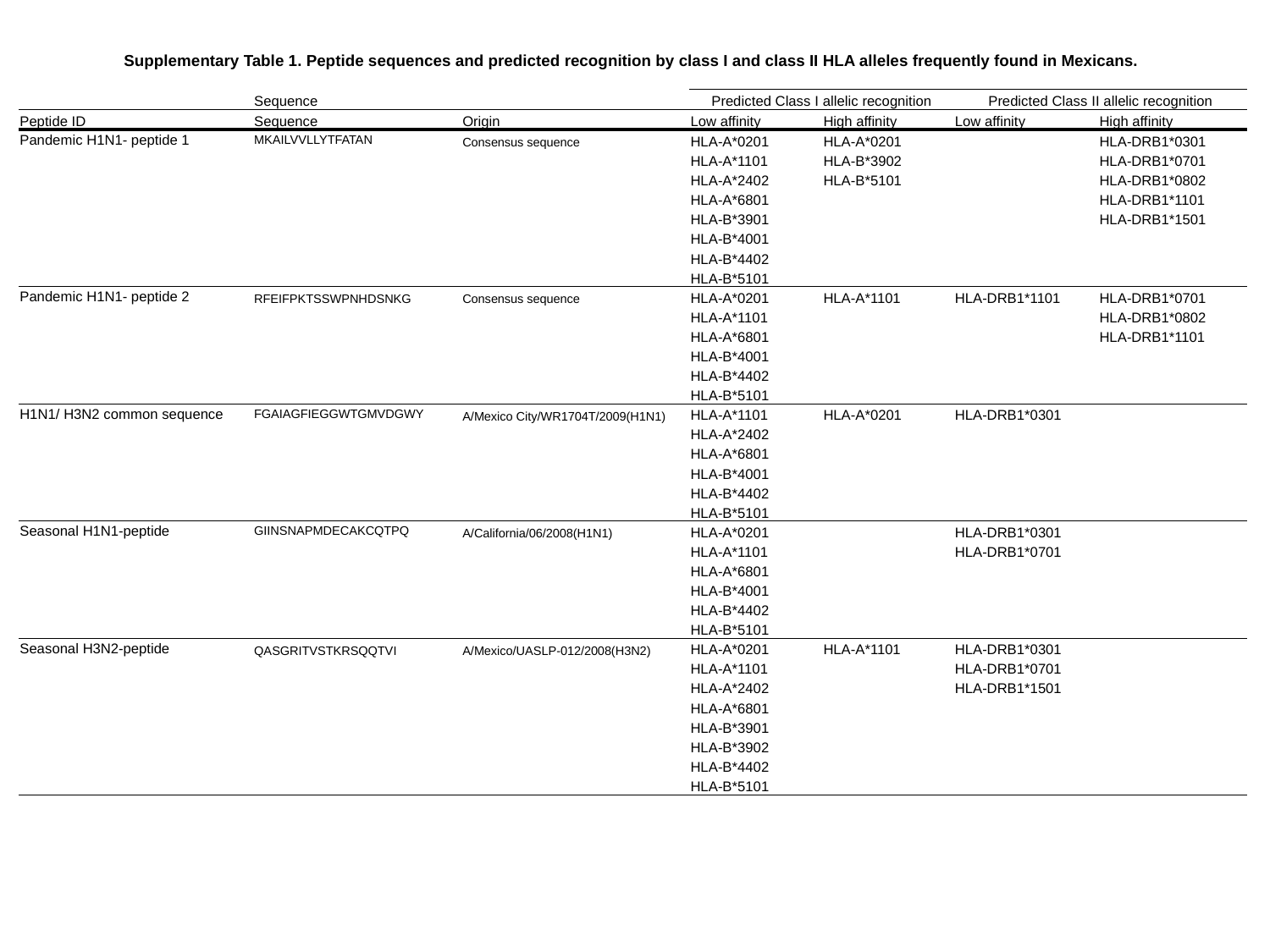

| Supplementary Table 1. Peptide sequences and predicted recognition by class I and class II HLA alleles frequently found in Mexicans. | | | | | | |
| --- | --- | --- | --- | --- | --- | --- |
| | | | | | | |
| | Sequence | | Predicted Class I allelic recognition | | Predicted Class II allelic recognition | |
| Peptide ID | Sequence | Origin | Low affinity | High affinity | Low affinity | High affinity |
| Pandemic H1N1- peptide 1 | MKAILVVLLYTFATAN | Consensus sequence | HLA-A\*0201 | HLA-A\*0201 | | HLA-DRB1\*0301 |
| | | | HLA-A\*1101 | HLA-B\*3902 | | HLA-DRB1\*0701 |
| | | | HLA-A\*2402 | HLA-B\*5101 | | HLA-DRB1\*0802 |
| | | | HLA-A\*6801 | | | HLA-DRB1\*1101 |
| | | | HLA-B\*3901 | | | HLA-DRB1\*1501 |
| | | | HLA-B\*4001 | | | |
| | | | HLA-B\*4402 | | | |
| | | | HLA-B\*5101 | | | |
| Pandemic H1N1- peptide 2 | RFEIFPKTSSWPNHDSNKG | Consensus sequence | HLA-A\*0201 | HLA-A\*1101 | HLA-DRB1\*1101 | HLA-DRB1\*0701 |
| | | | HLA-A\*1101 | | | HLA-DRB1\*0802 |
| | | | HLA-A\*6801 | | | HLA-DRB1\*1101 |
| | | | HLA-B\*4001 | | | |
| | | | HLA-B\*4402 | | | |
| | | | HLA-B\*5101 | | | |
| H1N1/ H3N2 common sequence | FGAIAGFIEGGWTGMVDGWY | A/Mexico City/WR1704T/2009(H1N1) | HLA-A\*1101 | HLA-A\*0201 | HLA-DRB1\*0301 | |
| | | | HLA-A\*2402 | | | |
| | | | HLA-A\*6801 | | | |
| | | | HLA-B\*4001 | | | |
| | | | HLA-B\*4402 | | | |
| | | | HLA-B\*5101 | | | |
| Seasonal H1N1-peptide | GIINSNAPMDECAKCQTPQ | A/California/06/2008(H1N1) | HLA-A\*0201 | | HLA-DRB1\*0301 | |
| | | | HLA-A\*1101 | | HLA-DRB1\*0701 | |
| | | | HLA-A\*6801 | | | |
| | | | HLA-B\*4001 | | | |
| | | | HLA-B\*4402 | | | |
| | | | HLA-B\*5101 | | | |
| Seasonal H3N2-peptide | QASGRITVSTKRSQQTVI | A/Mexico/UASLP-012/2008(H3N2) | HLA-A\*0201 | HLA-A\*1101 | HLA-DRB1\*0301 | |
| | | | HLA-A\*1101 | | HLA-DRB1\*0701 | |
| | | | HLA-A\*2402 | | HLA-DRB1\*1501 | |
| | | | HLA-A\*6801 | | | |
| | | | HLA-B\*3901 | | | |
| | | | HLA-B\*3902 | | | |
| | | | HLA-B\*4402 | | | |
| | | | HLA-B\*5101 | | | |
| | | | | | | |
